# Commentary: XBP-1 Is a Cell-Nonautonomous Regulator of Stress Resistance and Longevity

**DOI:** 10.3389/fnagi.2016.00182

**Published:** 2016-08-03

**Authors:** Gabriela Martínez, Claudia Duran-Aniotz, Felipe Cabral-Miranda, Claudio Hetz

**Affiliations:** ^1^Center for Geroscience, Brain Health and MetabolismSantiago, Chile; ^2^Biomedical Neuroscience Institute, Faculty of Medicine, University of ChileSantiago, Chile; ^3^Program of Cellular and Molecular Biology, Institute of Biomedical Sciences, University of ChileSantiago, Chile; ^4^Center for Integrative Biology, Universidad MayorSantiago, Chile; ^5^Buck Institute for Research on AgingNovato, CA, USA; ^6^Department of Immunology and Infectious diseases, Harvard School of Public HealthBoston, MA, USA

**Keywords:** aging, proteostasis cell stress and aging, unfolded protein response (UPR), cell-nonautonomous, proteostasis deficiencies, protein misfolding and disease, protein misfolding disease

The life expectancy in the world's population is increasing, highlighting the need of better understanding of the cellular and molecular pathways that drive the aging process. Because aging is the major risk factor to develop neurodegenerative conditions such as Alzheimer's and Parkinson's disease, the number of patients affected is constantly increasing, representing a major social and economic problem. Importantly, abnormal protein aggregation is a transversal pathological event of most aging-related brain diseases, suggesting that the ability of neurons to handle alterations in the proteome is specifically altered (Kaushik and Cuervo, 2015). Several hallmarks of aging have been identified at the cellular and molecular level (Lopez-Otin et al., 2013; Kennedy et al., 2014), highlighting alterations in protein homeostasis or proteostasis. In fact, studies in simple model organisms indicate that the buffering capacity of the proteostasis network (PN) is reduced during aging (Douglas and Dillin, 2010; Mardones et al., 2015). The PN can be decomposed in different interrelated sub-networks including mechanisms responsible for protein synthesis, translation, folding, trafficking, quality control, secretion, and degradation (Balch et al., 2008). Sustained dysfunction of one or more components of the PN may translate into cell dysfunction and even proteotoxicity (Figure 1).

**Figure 1:**
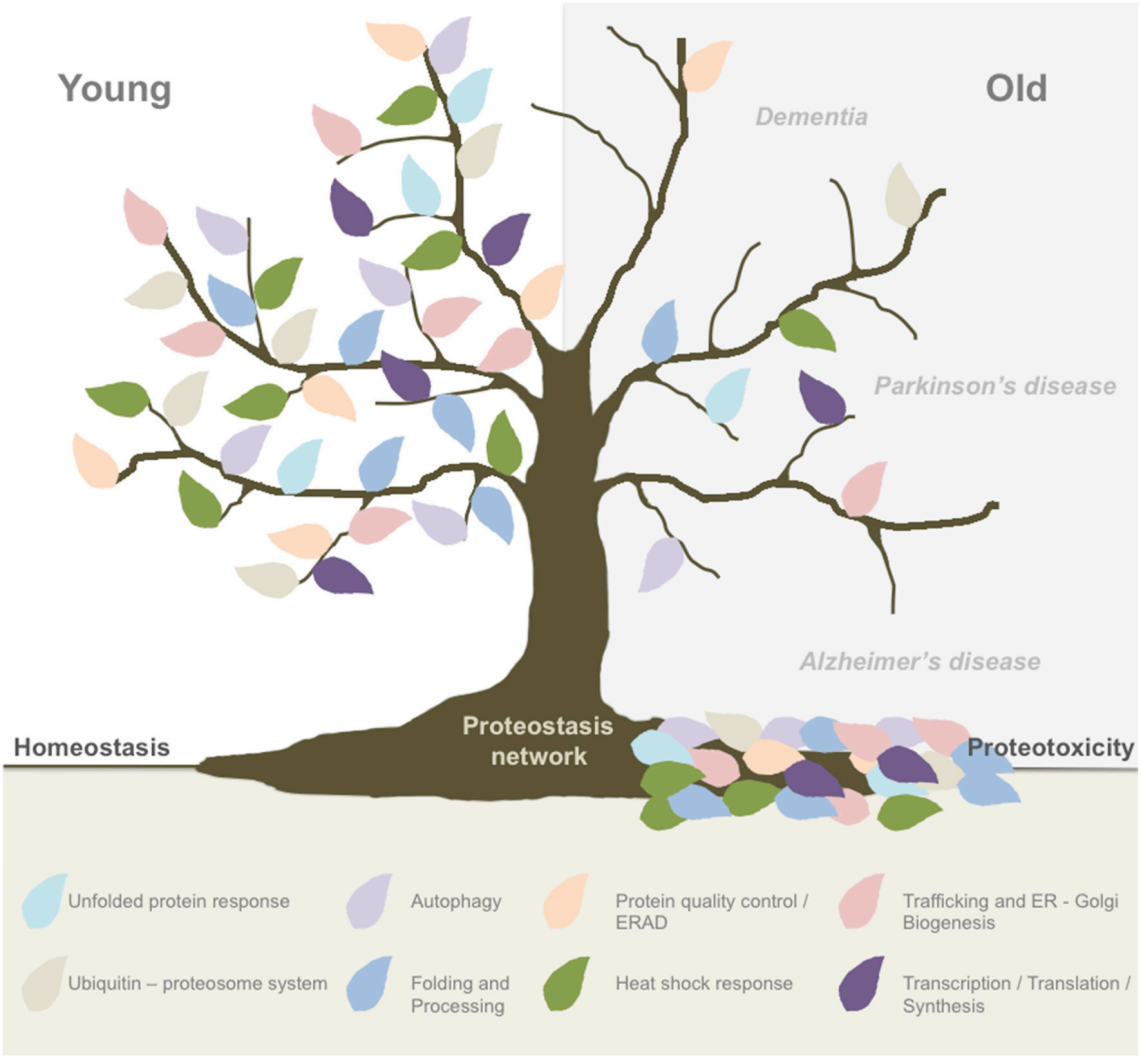
**Global proteostasis network impairment during aging**. Aging is the main risk factor to develop most neurodegenerative conditions and new evidence has pointed out to a progressive decline in the buffering capacity of the proteostasis network (PN) to handle cellular stress. The PN is formed by different interrelated sub-networks including mechanisms responsible for protein translation, folding, synthesis, protein quality control, trafficking, secretion, and degradation (ERAD, proteasome, autophagy). Proteostasis breakdown during aging may result in proteotoxicity and the development of neurodegenerative diseases such as Alzheimer's and Parkinson's disease.

Around 30% of the total proteome is synthetized at the endoplasmic reticulum (ER), an essential compartment involved in calcium handling, lipid synthesis among other functions. Different physiological and pathological stimuli can alter the function of this organelle, resulting in the accumulation of misfolded proteins. Importantly ER stress has been proposed as a central driver of several neurodegenerative conditions (Hetz and Mollereau, 2014). ER stress triggers the activation of the unfolded protein response (UPR), a central homeostatic pathway that orchestrates cells adaptation (Hetz et al., 2015). Studies in *Caenorhabditis elegans* and rats indicate that the activity of the UPR is drastically ablated during aging (Paz Gavilan et al., 2006; Naidoo et al., 2008; Ben-Zvi et al., 2009; Gavilan et al., 2009; Taylor and Dillin, 2013). The UPR is mediated by three main stress sensors located at the ER membrane including ATF6, PERK, and IRE1 (Ron and Walter, 2007). In brief, activation of IRE1 controls to the expression of the transcription factor XBP1s, leading to the upregulation of genes related with protein quality control, folding, ERAD, among other targets (Hetz et al., 2015). PERK phosphorylates eIF2α; inhibiting the translation of proteins into the ER, in addition to induce the expression of the transcription factor ATF4 regulating genes involved in the antioxidant response, amino acid metabolism and folding. Under irreversible ER stress ATF4 is essential to trigger apoptosis. ATF6 encodes a transcription factor in its cytosolic domain that upon processing is realized to control gene expression. Altogether, the activation of the UPR enforces adaptive mechanisms to sustain proteostasis or trigger cell demise when protein misfolding cannot be mitigated determining cell fate.

Several studies in model organisms have uncovered the significance of UPR signaling to the aging process. IRE1 is the only ER stress sensor expressed in yeast and contributes to lifespan extension (Labunskyy et al., 2014), consistent with the fact that UPR activation in this organism is a relevant feature involved in the health span control triggered by caloric restriction (Choi et al., 2013). Similarly, genetic modifications that enhance the activity of the UPR improve replicative lifespan in *Saccharomyces cerevisiae* (Cui et al., 2015). Studies in *C. elegans* demonstrated that ablating the expression of XBP1 reduces life expectancy, associated with altered FOXO and insulin/IGF-1 signaling, a canonical aging pathway (Henis-Korenblit et al., 2010). Importantly, another report indicated that the ectopic expression of XBP1s in neurons has a significant effect in increasing lifespan in *C. elegans* (around 30%), representing one of the strongest aging modulator described so far in this specie (Taylor and Dillin, 2013). In *D. melanogaster*, the occurrence of ER stress and chronic inflammation alters the stem cell pool in the gut, affecting intestinal homeostasis during aging (Wang et al., 2014). Unexpectedly, a recent study indicated that chronic PERK signaling limits lifespan by controlling intestinal homeostasis, having important consequences to organismal health (Wang et al., 2015). In mammals, it was reported that the capacity to response to ER stress and activate IRE1 is attenuated in macrophages during aging, increasing the susceptibility to apoptosis (Song et al., 2013). Accordantly, aged rats present more pro-apoptotic UPR components as opposed to adaptive mediators such as BIP, calnexin, and PDI after ER stress induction (Paz Gavilan et al., 2006; Naidoo et al., 2008). In contrast, during the aging process B cells, osteoclasts, adipocyte tissue, the retina, and muscle experience elevated levels of ER stress and UPR activation (Chalil et al., 2015; Ghosh et al., 2015; Lenox et al., 2015; Baehr et al., 2016; Kannan et al., 2016). These observations suggest that aging maybe associated with accumulative damage to the ER rather than an attenuation of UPR responses. However, the role of ER proteostasis impartment in mammalian aging needs to be functionally defined.

The UPR is emerging as a key player in the integration of systemic responses to handle proteostasis alterations at the whole organism, governed by the central nervous system (Sun et al., 2012; Taylor and Dillin, 2013). In addition to regulate the intrinsic capacity of the cell to respond to ER stress, activation of IRE1 in neurons engages an organismal reaction to promote stress resistance and longevity on a cell-nonautonomous manner (Taylor and Dillin, 2013). Interestingly, the activation of XBP1s in neurons *per se* was irrelevant to sustain organismal homeostasis, suggesting that the nervous system operates as a global adjustor of proteostasis, where the effectors in terms of enforcing aging resistance operate in the periphery, highlighting the intestine. Importantly, other studies have shown a similar mode of control for the heat shock response and the innate immunity in *C. elegans* (reviewed in Mardones et al., 2015). Similarly, in flies activation of PERK engages cell-nonautonomous responses in the gut during aging (Wang et al., 2015). The concept cell-nonautonomous UPR was recently validated in mammals, where the expression of XBP1s in the hypothalamus propagates signals to the periphery (i.e., the liver) to adjust energy metabolism (Williams et al., 2014). However, the specific mechanism of proteostasis control in mammals and the neuronal circuits mediating the propagation of UPR signals between cells remain to be determined. Importantly, in *C. elegans* the propagation of ER stress signals to the periphery depends on neurotransmitters, suggesting that signaling mechanisms may mediate the activation of UPR-like responses in the targeted tissue probably on a stress-independent manner (Taylor and Dillin, 2013). In this line, we recently reported that XBP1s has a novel function in controlling synaptic plasticity and behavior in mammals, where growth factors like BDNF can engage the pathway (Martinez et al., 2016).

Although several studies are placing the ER PN as a relevant adjustor of organismal aging in several species, its actual impact to human aging remains to be established. Many important questions need to be solved in this emerging field: Why is the UPR buffering capacity attenuated during aging? How does the nervous system control organismal proteostasis? Is there a connection between ER stress and aging in protein misfolding disorders affecting the nervous system? Can we exploit the control of cell-nonautonomous UPR as a therapeutic strategy to delay aging? Importantly, recent studies suggest that oxidative damage could directly modify UPR stress sensors, ablating adaptive responses (Nakato et al., 2015). In addition, the redox status of the ER is altered during aging in *C. elegans*, suggesting that intrinsic physiological alterations to this subcellular compartment may underlay the reduced capacity of the pathway to handle proteostasis alterations when cells get old (Kirstein et al., 2015). Several novel drugs are available to fine-tune the UPR and reduce ER stress levels (Hetz et al., 2013), which promises new avenues to intervene brain aging which may reduce the risk to develop neurodegenerative diseases, improving health span.

## Author contributions

GM: conceptualization, editing, and writing of the manuscript. CD: editing of manuscript, FC: editing of manuscript. CH: conceptualization, editing, and writing of the manuscript.

### Conflict of interest statement

The authors declare that the research was conducted in the absence of any commercial or financial relationships that could be construed as a potential conflict of interest.
